# Expanding equality? Institutional differentiation and reform in the access to higher education—the case of quota policies at USP

**DOI:** 10.3389/fsoc.2026.1790688

**Published:** 2026-05-26

**Authors:** Murillo Marschner Alves de Brito

**Affiliations:** Department of Sociology, Universidade de Sao Paulo, São Paulo, Brazil

**Keywords:** differentiation, educational stratification, higher education, USP, FUVEST

## Abstract

The central problem addressed in this article concerns a foundational issue in the research agenda on educational stratification, namely the association between social origins and educational destinations. A large body of recent literature in Brazil has focused on (A) changes in the mechanisms of access to public universities, and (B) the effects of the expansion of higher education on opportunity structures and social selectivity in access to tertiary education. Building on this research agenda, this article analyses access to public higher education, focusing more specifically on the case of the University of São Paulo (USP), which has implemented a significant set of changes in its admission mechanisms. Using socioeconomic and academic performance data on applicants, we estimate models with admission as the dependent variable. The results indicate that the university’s quota policy has fostered processes of inclusion primarily among students from the public school sector, although similar effects are also observed among black, pardo, and native students. Characterized by patterns of horizontal stratification in access, the implementation of the policy has established distinct opportunity structures through differentiation, contributing to a reversal of long-standing trends of social inequality reproduction in access to USP.

## 1 Introduction

The debate on educational stratification has increasingly turned to higher education as an object of analysis, given the scope and recurrence of expansion processes at this educational level across a wide range of countries in recent decades. The relationship between expansion, educational reforms, and inequality of opportunity has been central to this body of work, and international evidence underscores the importance of the institutional forms through which higher education expansion and reform take place, as well as their role in shaping parameters of horizontal stratification in access to this level of education. The Brazilian case is no exception: processes of higher education expansion in the country have been structured around reforms and public policy initiatives whose intended democratizing effects have been assessed by the specialized literature, which has produced evidence of processes of inclusion and democratization of access, particularly within the public sector.

This article intends to constitute (a) a contribution to the literature on educational stratification by discussing how processes of institutional diversification and differentiation (Neves, 2003; Sampaio, 2014; Prates and De Oliveira Barbosa, 2015) and parameters of horizontal stratification (Lucas, 2001, 2009; Mont’Alvão Neto, 2016; Carvalhaes and Ribeiro, 2019) define heterogeneous conditions for the establishment of opportunity structures in access to higher education in a context of reform in access mechanisms at this educational level, through an analysis of recent developments in the case of the University of Sao Paulo (USP) admission process—one of the largest in the country; and (b) an analysis of the effectiveness of recent reforms in democratizing access to USP, by mobilizing the theoretical and methodological framework of the educational stratification research agenda to assess the effectiveness of the recent implementation of policies aimed at democratizing access to USP, through an examination of the dynamics of association between social origins and chances of admission in the FUVEST selection process.

The relationship between educational expansion and the equalization of opportunities has long been addressed in the international literature (Blossfeld and Shavit, 1993; Raftery and Hout, 1993) and has more recently been mobilized to analyze the relationship between higher education expansion and inequalities of educational opportunity from a comparative international perspective (Arum et al., 2007). Case studies documenting specific educational reforms in access to higher education in national contexts have been produced (Ayalon et al., 2008; Reimer and Pollak, 2010; Boliver, 2011; Triventi, 2013; Iannelli et al., 2018; Schindler and Bittmann, 2021), as well as analyses of the Brazilian case, in which can be identified both the expansion of the higher education sector in terms of the number of students and institutions, diversification and differentiation at the systemic level, and the implementation of reforms aimed at changing access mechanisms, particularly regarding public higher education institutions (Rossetto and Gonçalves, 2015; Carvalhaes and Ribeiro, 2019; Tomás and Souza Silveira, 2021; Senkevics et al., 2022; Almeida et al., 2024).

In general terms, existing analyses demonstrate how different institutional arrangements result in distinct opportunity structures depending on the principles guiding reforms in access, while simultaneously highlighting the persistence of well-known parameters structuring opportunities even in contexts of expansion—such as gender, ethno-racial identity, socioeconomic status, proficiency, and social origin (parents’ or guardians’ occupation and educational attainment). However, almost without exception, (1) the literature develops analyses of processes of expansion, diversification, and differentiation at a systemic level (examining the set of institutions at the national level), leaving the specificities of these processes at the institutional level largely unexplored; (2) moreover, in most cases—particularly in the Brazilian context—this literature has focused exclusively on the population that enters higher education (as opposed to the relationship between the eligible population and those who are effectively admitted), which obscures the opportunity structure of access and weakens assessments of changes toward its democratization; (3) the documentation of processes of horizontal stratification in access remains incipient in the Brazilian case, since in most studies the evidence derives from household surveys with sampling designs that do not allow for detailed analyses by fields of study and careers.^1^ The research effort documented here seeks to contribute to advancing the Brazilian literature by addressing these three points, while also highlighting the relevance of case-study analyses for identifying the effects of educational reforms—specifically reforms in access mechanisms to public higher education—on general parameters of social stratification in access. By focusing on within-institution differentiation of access mechanisms, this article advances the literature by showing how institutional reforms reshape opportunity structures not only across systems, but also within institutions.

## 2 Expansion, differentiation and inclusion? Educational stratification in higher education

### 2.1 The debate on educational stratification

Processes of educational expansion have long been an object of analysis in the literature on educational stratification, which aims to understand how relationships between individuals’ social origins, educational attainment and destinations are established. A basic assumption of this literature is that the less unequal a society is, the less dependent individuals’ educational achievements will be on their social origins. In this sense, a central issue for understanding the evolution of processes of democratization of opportunities in modern societies lies in grasping the effects of expanding access to education over inequalities of educational opportunities, given that education constitutes a fundamental mechanism in the definition of class structures and social hierarchies.

The expansion of access to basic education through modernization has been extensively examined by a body of literature interested in understanding whether such expansion has promoted a reduction in class inequalities in the access to different educational levels. The accumulation of international empirical evidence has established a number of propositions regarding this relationship. Among the most influential are the propositions of persistent inequalities (Mare, 1981; Blossfeld and Shavit, 1993), which suggests that expansion processes do not tend to reduce class inequalities across cohorts, and the maximally maintained inequality (MMI) hypothesis (Raftery and Hout, 1993), which proposes that class inequalities in access to a given educational level tend to decline only when educational expansion reaches a point of saturation among the most privileged classes.

The key innovation of these propositions lies in questioning the assumption that educational expansion in itself leads to a reduction in class inequalities in the access to different educational levels: in an initial phase, expansion tends to increase educational inequalities, which are only reduced later through saturation, while simultaneously being displaced to higher educational levels. This type of interpretation became known as a “vertical” reading of inequalities, insofar as it seeks to understand the dynamics of class inequality in access to different educational levels based on models of educational progression that focus on individuals’ educational attainment, measured by the highest level of education achieved.

Such a framing of the relationship between expansion and inequality was subject to criticism in the early 2000s, based on the insufficiency of indicators of educational progression—those that simply capture access—to demonstrate parameters of class inequality within a segmented educational system (Breen and Jonsson, 2000). The effectively maintained inequality (EMI) hypothesis (Lucas, 2001) is the most influential of these critical formulations, bringing to the center of interpretation the idea of segmentation in educational trajectories. It posits that even when elites reach saturation in access to a given educational level, they seek advantages by entering more privileged segments of the system. In doing so, they differentiate themselves from other social groups and secure comparative advantages in their educational progression processes. Accordingly, analyses of the relationship between educational expansion and inequalities of opportunity that fail to consider the segmentation of educational systems into more and less privileged trajectories tend to underestimate class effects on educational progression—effects that become increasingly salient as expansion advances across educational levels. This type of interpretation has come to be known as a “horizontal” reading of inequalities, insofar as it refers to distinctions within educational systems that are observable at the same educational level.

In the early 2000s, several countries in the Global North had already made substantial progress in the expansion of basic education (primary and secondary), processes that had been extensively analyzed through the lens of educational progression models, and the literature turned more systematically to the analysis of higher education expansion. This third generation of studies on educational stratification builds on earlier developments to construct interpretations of the relationship between higher education expansion and class inequalities. The influence of arguments concerning segmentation and the prominence of horizontal inequalities is a defining feature of scholarship from this period, given that the diversity of educational trajectories—particularly across types of institutions and fields of study—distinguishes the institutional forms of higher education from those of other educational levels. As a result, a body of literature emerged with a strong interest in examining the ways in which national expansion processes unfold in terms of their capacity for inclusion and the extent of their institutional differentiation (Arum et al., 2007).

An established argument in this literature holds that the institutional form of expansion gives rise to distinct parameters of inequality of opportunity: more institutionally diversified systems tend to offer less unequal opportunities, with class inequalities manifesting themselves through segmentation—whether institutional or by field of study. This perspective has framed expansion as a process of inclusion grounded in institutional differentiation at the systemic level, increasing access through lower-prestige institutions as higher education systems expand and become more diversified. This research agenda thus points to the tension between inclusion and segmentation as the central axis of interpretations regarding the effects of higher education expansion on inequalities in access, examining evidence on the articulation between vertical inequalities (in access) and horizontal inequalities (in access by type).

It is with this conceptual framework on inequalities of educational opportunity that this article seeks to engage, particularly its more recent developments in the interpretation of higher education expansion processes, an area in which there is a substantial accumulation of international scholarship, including studies on Brazil. Two broad theoretical questions guide our analysis: first, the relationship between social origins and educational destinations—which represents the degree of democratization of educational opportunities; and second, the relationship between the institutional forms of expansion and their effects on educational opportunities—which concerns the capacity of institutionally driven reforms to shape the structure of educational opportunities.

### 2.2 Educational stratification, expansion and reforms in higher education: international research

The literature on higher education expansion and its relationship to inequalities of educational opportunity is highly diverse and draws on evidence produced through the analysis of a large number of national cases. While countries undoubtedly differ greatly in the levels of access their populations have to higher education, expansion processes are observable across many of them, and the specialized literature has identified both common patterns and distinctive features in these processes.

The distinction between unified systems (based on state provision of higher education through large academic institutions with a propaedeutic orientation), binary systems (state provision through large university institutions alongside vocational and technical-scientific institutions), and diversified systems (public and private provision through a more heterogeneous set of institutional types—academic, technical-scientific, vocational, community-based, etc.) constitutes a widely used typological framework in this literature (Arum et al., 2007), and has guided the analysis of numerous national cases and international comparisons. The most general hypothesis explored is that expansion grounded in more diversified institutional arrangements—that is, diversified systems—tends to promote greater inclusion, but also greater segmentation in access, with a higher proportion of individuals from less privileged classes entering the system while being disproportionately concentrated in certain types of institutions and fields of study, generally those of lower prestige.

Among national case studies, the United States is perhaps the most extensively documented. Characterized as a diversified system, it reinforces patterns already well established for countries of the Global North, namely that inequality in access to higher education declined over the course of the twentieth century, even though the effects of parental higher education on access chances have remained stable over time. At the same time, the process of access expansion is marked by a U-shaped trend in the effect of income, explained by the rising costs of higher education and by overall increases in income inequality among families of origin (Bernardi et al., 2018). Expansion has also been consolidated through processes of horizontal stratification, both across different types of institutions and across fields of study, increasingly grounded in “meritocratic” mechanisms—most notably standardized tests and engagement in extracurricular activities—for the allocation of vacancies, whose relative weight varies depending on levels of competitiveness (across cohorts and fields), particularly in periods of high competition (Posselt et al., 2012). These micro-foundations of maximally maintained inequality would sustain a “vicious cycle of adaptation and exclusion” (Alon, 2009), favoring the persistence of class and racial inequalities in access to prestigious institutions.

The literature examining European countries is also extensive, and recent studies have questioned the persistence of class inequalities in the face of expansion processes at the basic educational levels (Breen et al., 2009). The expansion of higher education has largely been based on differentiation between three-year undergraduate programs (bachelor’s degrees) and five-year programs (master’s degrees), offered by different types of institutions within predominantly binary systems. This institutional form of expansion has, on the one hand, promoted increased access in countries with lower initial levels of participation and, on the other, led to the persistence or even strengthening of class effects on chances of access to different types of institutions and programs (Katrňák and Hubatková, 2022), particularly in countries where institutional differentiation is more consequential for occupational outcomes (Triventi, 2013).

In the case of European countries, the comparative literature is somewhat more developed, but there is also a substantial body of studies focused on national systems. Research on Germany—recurrently documented as a stratified binary system dividing vocational higher education and technological/academic higher education—has highlighted the importance of the effects of institutional forms of expansion on both horizontal and vertical stratification in access to higher education. Evidence from pre- and post-reunification cohorts shows the effects of system reforms in the former GDR, with an increase in inequality of educational opportunity after reunification following the alignment of the GDR system with that of the FRG within a unified national system (Betthäuser, 2019); the effects of institutional diversification of access mechanisms to higher education in a system strongly shaped by vocational segmentation (Schindler and Bittmann, 2021); and the persistence of significant effects of social origin on both vertical stratification (of access) and horizontal stratification (by fields of study and by institutions) as processes of expansion and differentiation advance (Reimer and Pollak, 2010). Similar patterns of horizontal and vertical stratification in access to higher education are observed in the French case, also a system that developed as a binary system during expansion, with the establishment of persistent class barriers in access to more prestigious institutions and careers (Duru-Bellat et al., 2008).

The literature also documents a body of evidence on the expansion process in the United Kingdom, interpreted through two key movements: an initial phase of expansion of lower-prestige institutions, creating a second tier relative to already established traditional institutions (in the 1970s), and a second phase of expansion emerging in the 1990s, which significantly increased access and has been interpreted as a process of higher education massification (Boliver, 2011). Vertical and horizontal stratification parameters throughout processes of expansion and differentiation in the UK were both maximally and effectively maintained; however, given the stability of class effects—particularly in patterns of field-of-study choices—the argument is that expansion was more inclusive than exclusionary, resulting in a system in which horizontal stratification processes did not become strongly established as mechanisms of social reproduction (van Van De Werfhorst et al., 2003), largely due to the expansion of access to more prestigious fields within less prestigious institutions (Iannelli et al., 2018).

But the literature on higher education expansion is not limited to the United States and European countries, allowing for advances in understanding class effects across a more diverse set of reform and expansion processes.

The Japanese case is characterized as an expansion with low levels of state investment—thus higher educational costs borne by families—and is strongly stratified by differentiation across types of institutions (Fujihara, 2023). With an institutional form heavily grounded in market provision, income inequality in access increased, even as institutional selectivity declined. In China, a massive expansion process characterized by rising schooling costs and by the expansion of urban vocational secondary education led to increased access levels but also to heightened inequalities, favoring students from more privileged backgrounds and accentuating regional inequalities, in line with the maximally maintained inequality hypothesis (Wang, 2021).

The Russian case (and that of the former USSR) has historically been interpreted as a case of a “bottleneck” in the transition to higher education, in which the expansion of upper-secondary completion far outpaced the opening of higher education vacancies (Gerber and Hout, 1995) during the Soviet period. This was followed by an increase in inequalities by social origin after the collapse of the USSR, with vocational education becoming a more common destination among working-class youth, thereby reducing overall access parameters to higher education during the process of political and economic liberalization (Gerber, 2000). More recent reassessments of the Russian case highlight the historical centrality of educational reforms in both the pre- and post-communist periods in shaping opportunity structures, such as the adoption of quotas targeting rural populations and the working classes during the Khrushchev period, and the marketization of higher education provision from the 1990s onward (Yastrebov, 2022), even as they document general trends toward declining stratification in access across cohorts born in the twentieth century.

The Israeli case is also extensively documented, particularly with regard to the effects of reforms in the 1990s aimed at increasing access levels—through the reduction of university eligibility requirements—and reducing inequalities between social classes and ethno-racial groups. These reforms reduced inequalities in access—even in the absence of saturation among privileged classes—but consolidated a system of horizontal stratification that incorporated working-class students and certain ethno-racial groups into less selective institutions, a process of differentiation that maintained stable class barriers in access to more prestigious academic-university institutions (Ayalon and Shavit, 2004; Ayalon et al., 2008; Alon, 2009).

Latin America also features a substantial body of studies on inequalities of opportunity in access to higher education. International comparisons have highlighted distinct trajectories between Latin American countries and those of the Global North, particularly the resurgence of inequalities during the economic crisis of the 1980s, which constrained families’ capacity to invest in education (Torche, 2010), at a time when higher education systems in the region had not yet undergone significant expansion. More recent studies have documented differences in the scope of the expansion processes, which have been less pronounced in Argentina—where expansion has been based on a binary system (Boniolo et al., 2025)—than in Brazil, Mexico, Chile, Colombia, and other countries characterized by more diversified higher education systems. In these cases, countries in which expansion began at lower levels of access (such as Brazil) experienced more pronounced declines in class inequalities, whereas in countries that started from higher access levels (such as Argentina, Mexico, and Chile) these declines were less marked, and class, gender, and ethno-racial barriers proved more resistant as expansion processes advanced.

In South America, the Brazilian case stands out from that of other countries for exhibiting the lowest levels of access, both at the outset and after the consolidation of higher education expansion processes (SITEAL, 2024), despite a substantial increase in the number of students at this level between the 1990s and the 2010s—a period during which the student population grew from 1.5 million to 8 million individuals. Patterns of stratification in access are extensively documented in the literature, and class and racial inequalities have long constituted major obstacles to educational trajectories in the country. The expansion of access to education more broadly has been interpreted through the lens of maximally maintained inequality (Silva and Hasenbalg, 2002; Ribeiro, 2011; de Brito, 2017), with a particular emphasis on racial inequalities in access to higher education (Fernandes, 2004), which increased even as access to primary education became universal and significant advances were made at the secondary level. This scenario began to change in the early 2000s, with the expansion of secondary education and the implementation of affirmative action policies and public student financing programs with class- and race-based criteria, which increased the chances of entering higher education for historically excluded groups (Marteleto et al., 2016) among cohorts of young people born in the 1990s. Reductions in access barriers at the primary and secondary levels, as well as declines in income inequality observed in the country—particularly during the 2005–2015 period—had democratizing effects, fostering access to higher education (Salata, 2018). Long-term trends documented in the literature suggest a clear decline in class effects (Salata et al., 2025). Nevertheless, the dual character of the Brazilian higher education system—which juxtaposes a minority of tuition-free public institutions with a large private sector—has generated parameters of horizontal stratification across different types of institutions and fields of study (Mont’Alvão, 2011), favoring access by white youth from privileged families to public institutions and more prestigious careers.

The Brazilian expansion process was accompanied by reforms in access mechanisms to public universities aimed at their democratization, implemented on a large scale from the early 2010s onward—most notably the adoption of racial and socioeconomic quota policies in student selection for these institutions, particularly through Law 12.711/2012, widely known as the “Quota Law.” This legislation reserved places for Black, pardo, and native students, as well as for students from public schools at the basic education level (primary and secondary). The law consolidated and extended to all federal universities a process that had been underway since the mid-2000s, namely the adoption of quota policies across public universities at all levels—municipal, state, and federal (Daflon et al., 2013). Concurrently, public financing policies facilitating access to the private sector were also expanded, through programs such as FIES and ProUni,^2^ likewise aimed at reducing economic, class, and racial barriers to higher education—predominantly private^3^ in the Brazilian case.

More recent scholarship has turned to documenting the effects of these reforms on access patterns in the Brazilian case and has recorded the establishment of horizontal stratification parameters. In terms of stratification by fields of study, class and gender inequalities—and to a lesser extent, racial inequalities—characterize the population that effectively gains access to higher education, channeling students from privileged families into more prestigious programs and into public institutions, while also maintaining gender segregation patterns observed in other national contexts (Carvalhaes and Ribeiro, 2019), with consequences for chances of higher education completion as well (Knop and Collares, 2019). This literature has also documented the consolidation of highly distinct and heterogeneous opportunity structures between the public and private sectors. The adoption of centralized systems for the allocation of places based on standardized tests (the ENEM),^4^ combined with quota policies in public universities, has increasingly linked opportunity structures of access in this sector to student performance, often interpreted as a mechanism for the transmission of class privilege. In the private sector, class inequalities are more pronounced, since access depends less on performance and more on families’ capacity to finance education (Senkevics et al., 2022). What emerges, therefore, are distinct access regimes between the public and private sectors, illustrating how the institutional design of the Brazilian system shapes compensatory advantages that may either reinforce or reduce inequalities of educational opportunity. In the Brazilian case, privileged students benefit from a double advantage: high performance increases their chances of admission to the public sector, while family resources compensate for lower performance by ensuring access to the private sector (Senkevics et al., 2024).

Segmentation in the public sector—characterized by institutions that integrate teaching, research, and extension, and by universities of different sizes and specializations—and in the private sector, which comprises universities, higher education centers, colleges, and other types of institutions that are predominantly teaching-oriented, constitutes a highly differentiated and heterogeneous system. To this complex and characteristically differentiated institutional configuration—established with the 1988 Constitution and implemented through the guidelines defined in the 1996 National Education Guidelines Law (LDB), which provided the normative foundation for the expansion of higher education in the country—were added, particularly from the mid-2000s onward, a set of institutional reforms aimed at promoting criteria intended to reduce inequalities of opportunity in access in both the public and private sectors. This is the broader institutional setting within which the case study examined in this article is situated: the University of São Paulo, one of the largest public higher education institutions in the country.

In this section, our objective was to demonstrate how discussions concerning the outcomes of educational reforms on patterns of inequality of opportunity—particularly in access to higher education in contexts of expansion—have mobilized the production of an extensive international literature. The institutional form assumed by expansion processes has been a central object of analysis within this research agenda, which informs the formulation of our research problem and the analysis of the case at hand. In the Brazilian context, system-level analyses have been produced in order to identify the general effects of reforms on opportunity structures, highlighting heterogeneity across segments (public and private) and across institutions, as well as the differentiation processes that have characterized the higher education system as a whole as mechanisms underpinning parameters of vertical and horizontal stratification. The contribution of this article lies in seeking in adding to this discussion an aspect that remains relatively underexplored in the literature: the analysis of how reform initiatives in access affect opportunity structures within institutions, promoting internal differentiation in stratification patterns. In order to better situate the case examined in this article, the following section presents a review of interpretations of the Brazilian expansion process, a description of nationally implemented policies, and an account of how these policies have, in recent periods, translated into reforms of access mechanisms at the University of São Paulo (USP), on which the empirical exercises presented here are based.

## 3 Expansion and reforms in Brazilian higher education

### 3.1 Public policies and the expansion of higher education in Brazil

The debate on expansion, differentiation, and stratification in access to higher education was already grounded in notions such as institutional differentiation and functional specialization in the early 2000s (Neves, 2003), at a moment when the complexity of an institutional arrangement increasingly marked by system diversification across types of institutions and programs/careers was being documented. With the approval of a new regulatory framework in 1997, the process of Brazilian higher education expansion gained momentum through the authorization for institutions to operate for profit. Supported as well by demographic factors, by advances in the incorporation of students into upper-secondary education, and by adjustments in educational trajectories at the basic levels, the 2000s and 2010s were marked by significant increases in the size of the higher education student population. During this period, the specialized literature advanced in characterizing institutional expansion and engaged in intensive debate over the democratizing effects of expansion, juxtaposing tendencies toward differentiation and isomorphism (Sampaio, 2014), and speculating about the potential emergence of radical credentialism^5^ (Prates and De Oliveira Barbosa, 2015) as a future outcome of an increasingly differentiated system.

But the Brazilian expansion process was also strongly shaped by the introduction of public policies aimed at democratizing access to higher education, directed at both the private and the public sectors, and grounded in goals set out in the National Education Plans (*Planos Nacionais de Educação*—PNEs) elaborated by the federal government—the first in 2001 and the second in 2014—focused on increasing coverage. The main initiatives to expand access within the private sector were the ProUni and the FIES, both policies designed to finance tuition at private institutions. ProUni is a policy that provides partial and full scholarships at private institutions to students who meet a set of socioeconomic criteria and performance requirements on the ENEM. In return, students are required to maintain certain levels of academic performance throughout their undergraduate studies, while participating institutions receive federal tax incentives and are simultaneously required to achieve satisfactory evaluations within the SINAES (National System for the Evaluation of Higher Education). FIES is a student loan policy, implemented in 2001, which underwent significant expansion and redesign in 2010. Access to this policy is granted to a broader segment of the student population, including those with higher income levels; however, it operates as a loan rather than a grant, and does not impose academic performance requirements.

In the public sector, two other initiatives stand out during this period. The first was Reuni,^6^ implemented in 2007, which provided additional resources to federal public universities in order to finance plans to expand their enrollment capacity through the creation of new programs and campuses, the hiring of faculty and staff, and the implementation of affirmative action and student retention policies. The implementation of Reuni enabled a significant expansion in the supply of public places at federal universities, which doubled over the period in which the policy was in effect (2007–2013). The second initiative directed at the public sector was the drafting and implementation of Law 12.711/2012, known as the “Quota Law,” which established procedures for the allocation of vacancies in federal institutions based on socioeconomic criteria, racial criteria, and prior educational trajectories within the public basic education system. The quota provisions set out in the law were to be implemented by federal institutions until, by 2016, they reached the threshold of 50% of admitted students coming from public schools, within which places would be further stratified according to socioeconomic and racial criteria. The implementation of this policy marked the culmination of a broader movement among public institutions in the country—both federal and state-level—that, since the mid-2000s, had been implementing different affirmative action policy experiences aimed at promoting the democratization of access for a range of population groups historically underrepresented in the public higher education system in Brazil (De Oliveira and Silva, 2017; Da Silveira, 2018; Pires et al., 2020).

From a systemic perspective, heterogeneity across institutions and programs has increased, with greater diversification of the student body occurring alongside the consolidation of hierarchical patterns in Brazilian higher education that reflect broader social hierarchies in access by organizational type, administrative category, and academic degree (Tomás and Souza Silveira, 2021). The increase in institutional heterogeneity and the diversification of student profiles contributed to the democratization of access, albeit with limitations that pointed to higher chances of entry into the private sector for youth from lower socioeconomic backgrounds, concentrated in lower-prestige programs and institutions (De Oliveira and Silva, 2017), and characterized by a regional concentration of provision (Da Silveira, 2018). Even so, the programs and fields that expanded the most during this period (such as law, civil engineering, and business administration) were precisely those that experienced the most pronounced reductions in class-based barriers to access (Roza, 2023).

Analyses of policies directed at the public sector have demonstrated processes of transformation in the profile of the population accessing public universities in Brazil. The introduction of quota policies in admission processes at federal public universities has increased the proportion of admitted students coming from public basic education, as well as of black, pardo, and native students, particularly in institutions that, prior to the adoption of the Quota Law, had low proportions of entrants with this profile (Senkevics and Mattioli Mello, 2019). The adoption of quota policies based on Law 12.711/2012 is mandatory among federal institutions, and case studies of some of these universities have documented such changes at the institutional level. As a rule, however, they also document the consolidation of patterns of segmentation by class and gender and the establishment of horizontal stratification across programs and careers, both at entry (Karruz, 2018) and in dropout and completion chances (da Costa and Picanço, 2020; De Paula et al., 2023). Indeed, the implementation of these policies occurred heterogeneously across institutions, many of which had already adopted quota arrangements prior to the Quota Law (Campos and Lima, 2025), although the law—combined with the adoption of a centralized place-allocation system (SISU)^7^—promoted a degree of homogenization of these initiatives among federal institutions. However, the Brazilian public higher education system is not composed exclusively of federal institutions, and the broader movement toward the adoption of quota policies reinforced such initiatives in some cases—such as at UERJ—and fostered them in others—such as at USP and Unicamp—leading to the adoption of quota policies within state-level public universities as well.

In the Brazilian case, the 2000s and 2010s were characterized by the expansion of higher education and by significant institutional changes in university access mechanisms, in both the private and public sectors. It is at this point that we situate the contribution of this article: considering the set of recent reforms—particularly those aimed at transforming access mechanisms to public institutions through the adoption of quota policies—we analyze their effects on access stratification at USP, a state-level public university that is therefore autonomous in defining its selection criteria with respect to the Quota Law. While USP has adopted access-democratization policies since 2007, it was only from 2019 onward that it began to implement quota policies.

### 3.2 Access democratization policies at USP

Founded in 1934, USP is one of the largest and most traditional public universities in the country and currently enrolls more than 58,000 undergraduate students, distributed across 325 undergraduate programs. The main selection mechanism on the institution is the entrance examination organized by FUVEST (a foundation affiliated with the university itself), through which approximately 70% of the available places in its undergraduate programs are allocated. Applicants are selected according to their overall performance in two types of examinations—a standardized test in the first phase of the selection process and a written exam in the second phase, which only applications approved in the previous phase are eligible to take. This is one of the largest university selection processes in the country, with more than 100,000 applications annually in recent years competing for approximately 8,000 places distributed across the institution’s undergraduate programs.

Inequality in access to USP has been investigated since the university began adopting access-democratization policies. The process of expansion and diversification of the university, which took place mainly from the 1960s onward, was characterized by the establishment of a departments and institutes that contrasted more traditional fields—a traditional professional pole, composed of more elitized careers offered in older institutes—with careers more oriented toward technical professional training and academic-scientific formation, in institutes and colleges established in more recent periods of expansion, particularly after the 1970s and during the expansion of higher education in the country in the 2000s—a scientific-academic and technical pole (Carlotto, 2018). Historically, patterns of access to programs within these poles have differed, being more socially selective in the courses and careers of the traditional pole compared to the scientific-academic and technical-professional poles, which have generally been more closely associated with processes of inclusion. Socially recognized as an elite and elitist institution, USP only began to implement access-democratization policies in the mid-2000s—initially in the form of performance bonuses in the selection process for historically excluded social groups, and later through the adoption of quota policies.

The first access-democratization initiative implemented by USP was INCLUSP,^8^ focused on applications from individuals who had completed their entire schooling in the public school system, through a mechanism known as the “Additional Scoring System,” implemented in 2007. In 2009, this initiative was incorporated into a broader bonus policy that increased the percentage added to the exam scores of students coming from public upper-secondary schools, the PASUSP. Between 2009 and 2018, PASUSP/INCLUSP was the access-democratization policy adopted by the university, progressively encompassing a broader target population. This occurred through the inclusion, in 2012, of students who had also attended the public sector at the primary education level, and through the implementation, in 2014, of a fixed bonus for applications from individuals who self-identified as black, pardo, and native (PPI). The bonus applied to exam scores also increased over this period, rising from a maximum of 3% in 2007 to a maximum of 20%, introduced in 2014 and remaining in effect until 2017.

In 2018, some of the most significant reforms took place, involving major changes in the mechanisms for allocating places in the FUVEST entrance examination. PASUSP/INCLUSP was discontinued, and the FUVEST exam adopted a quota policy, establishing a process of differentiation by type of application: Open Competition (AC); Affirmative Action (EP)—reserved for students from public basic education; and Affirmative Action (PPI)—reserved for self-identified black, pardo, and native students from public basic education.^9^ At application, candidates chose their type of application, and competition for vacancies take place at the level of programs and courses within type of application (AC and EP-PPI). These changes implemented initiatives based on Resolution GR-7373 of July 10, 2017,^10^ which established a quota of 40% of places reserved for students from public schools for the 2019 entrance, increasing to 45% in 2020 and reaching 50% from 2021 onward (without reference to racial identification criteria). This reform thus represents a turning point in the trajectory of access-democratization policies at USP, with the incorporation of quota policies into the FUVEST entrance examination. The target of reserving 50% of places for students from public schools in 2021 marks the end of the reform implementation process, after which the university’s selection mechanisms remained largely unchanged.

There is no doubt that this was a dynamic period in terms of institutional reforms in access, aimed at promoting transformations that can be appropriately interrogated through a body of scholarship concerned with understanding the association between social origins and educational destinations. A normative analysis of these reforms makes it possible to document how the expansion of the university has been articulated with social demands regarding entrance, both in terms of its institutional differentiation (the creation of new programs, campuses, types of application, etc.) and in terms of its interaction with demographic trends (expressed, for instance, in the applicant/admission ratio). From this point forward, we are interested in advancing our understanding of how these reforms affected the opportunity structure for access to the university.

The study of the USP case documents how the initial access-democratization initiatives—based on score-bonus mechanisms—promoted increases in the chances of inclusion for individuals coming from public basic education with lower socioeconomic status than those who had previously been admitted. However, in the absence of clearly defined inclusion targets, the effects of the bonus were observed in a highly heterogeneous manner across programs, favoring the entry of policy beneficiaries primarily into lower-prestige programs, to the detriment of more competitive careers within the traditional professional pole (Piotto and Nogueira, 2016). Analyses of changes in the profile of the student population during the reform period suggest a process of university democratization between 2000 and 2020, with increases in the proportion of students coming from public basic education and of black and pardo students, during a period marked by the implementation of affirmative action policies, the intensification of vacancies expansion, and growth in university investment in student retention policies (Almeida et al., 2024).

However, there are still no studies aimed at analyzing the effects of these reforms on the opportunity structure generated by the selection process, particularly with respect to the implementation of quota policies at the university. These reforms promoted a process of internal differentiation within the institution regarding admission mechanisms, with the explicit goal of enhancing the chances of specific population groups—black, pardo, and native students, as well as students coming from public basic education. We argue that mobilizing the theoretical framework of the educational stratification research agenda—particularly recent contributions addressing the relationship between higher education expansion and inequalities of educational opportunity—combined with the body of work examining the effects of recent reforms in access to public higher education in Brazil, provides a solid foundation for analyses of the capacity of these initiatives to reduce inequalities in chances of access to university education. This is the contribution we seek to present in this article, in which we analyze chances of admission to USP and how these chances are conditioned by the socioeconomic characteristics and social origins of individuals who applied to the selection processes during the period of the quota policy adoption, between 2019 and 2023.

## 4 Hypothesis

Considering the construction of the problem of inequality of educational opportunity as an issue concerning the relationship between social origins, socioeconomic characteristics, and educational outcomes, we start from the basic hypothesis that admission to USP through the FUVEST entrance examination is conditioned by these characteristics, either directly or indirectly (mediated by performance in the selection process).

Taking into account assumptions regarding the relationship between higher education expansion and inequalities of educational opportunity—particularly the assumption concerning the relationship between educational reforms and opportunity structures—and the specific intentions of USP’s reforms to promote the inclusion of particular groups of applicants, we propose testing a hypothesis that represents the policy’s stated intentions:

a Inclusion Hypothesis—the adoption of quota policies increases the chances of admission for applications from individuals coming from public basic education, as well as for black, pardo, and native individuals.

However, the study of reforms in the institutional organization of higher education systems throughout expansion processes in international contexts suggests—particularly in diversified systems—the emergence of patterns of horizontal stratification in access. With the aim of testing potential effects of this type generated by the reforms implemented in the selection process for USP, we propose:

b Segmented Inclusion Hypothesis—the adoption of quota policies increases chances of admission in a heterogeneous manner across fields of study, with more pronounced effects in fields of lower social prestige and less pronounced effects in fields of higher prestige.

Still within the terms of the discussion on reforms and opportunity structures, and considering what we identify as a gap in this literature—namely, that little is still known about how processes of internal differentiation within institutions are established and how such differentiation processes affect opportunity structures—we analyze the adoption of the quota policy at USP as the establishment of a process of differentiation in the institution’s admission mechanisms. Accordingly, we test whether the opportunity structure of access across different types of application is significantly distinct, based on the following proposition:

c Internal Institutional Differentiation Hypothesis—socioeconomic and social origin characteristics (both direct and indirect) will condition chances of admission in distinct ways and with different intensities, depending on the type of application (AC/EP–PPI).

With this set of hypotheses, we seek to develop empirically grounded considerations regarding the effects of recent reforms in the selection process at USP; to foster a dialogue between the effects of these reforms at USP and the evidence on the effects of reforms in the Brazilian federal public system—with which they share a certain degree of similarity, at least in terms of format (quota policies) and broader political intentions (the democratization of access); and to contribute to a broad international literature interested in analyzing the effects of different institutional forms of educational reform on the structure of opportunities for access to higher education.

## 5 Data and methods

The primary source of information consists of databases derived from socioeconomic questionnaires responded by individuals at the time of application. This is a structured instrument that collects socioeconomic and educational characteristics of applicants annually. These data are not publicly available; therefore, following a formal request, FUVEST provided us with individual-level microdata for the years 2019 through 2023. To the data obtained from the socioeconomic questionnaire, we incorporated information on (a) the field, career, and program chosen; (b) outcomes of the selection process—namely performance on the standardized first-phase test and the admission outcome; and (c) the type of application in the selection process (AC/EP–PPI). This set of informations allows us to delineate two distinct populations—the population of individuals who submitted applications and, among them, the subgroup of individuals who were admitted—thereby characterizing the educational transition of interest in this study.

The dependent variable of the study is therefore admission in the selection process. Competition takes place among applicants that selected the same career, within each type of application (AC/EP–PPI).^11^ In order to represent the set of socioeconomic and social-origin conditions of applicants, we standardized a set of variables across years: sex (female = 1); race (black/pardo/native = 1); per capita household income (in LN(BRL)); higher education in the family (at least one parent with higher education = 1); labor market participation (40 h or more per week); public basic education (basic education mostly in the public sector = 1); and academic performance (score on the first-phase test—90-point scale). These variables are used for the estimation of the logistic model

$$\ln(P(Y=1)/(1-P(Y=1)))=\beta_{0}+\beta ₁X₁+\beta ₂X₂+\ldots +\beta_{n}X_{n}$$

where:

- *P*(*Y* = 1) is admission probability;
- *ln*(*P*(*Y* = 1)/(1 − *P*(*Y* = 1))) is the logit function;
- *β*₀ is the model’s intercept;
- *β*_1_, *β*_2_,… *β_n_* are regression coefficients for the vector of covariates *X*_1_, *X*_2_,… *X_n_*.

We carried out multiple estimations of this model in order to cover the three main analytical cuts: (a) the total population of applicants; (b) groups of applicants by field (STEM; Health and Natural Sciences—HNS; Humanities and Arts—HUM); and (c) groups of applicants by type of application (AC/EP–PPI), totaling six model estimations for each year. With this specification, we aim to test the three working hypotheses outlined above and to identify temporal trends in the variation of the determinants of admission following the implementation of quota policies that differentiate access routes. The following section presents the descriptive and inferential results of the implementation of the proposed analytical design.

## 6 Results

### 6.1 Descriptive statistics

The FUVEST entrance examination is one of the largest university selection processes in the country. Over the period analyzed in this study (2019–2023), there were a total of 492,795 applications with complete information available for the analyses.^12^ Table 1 presents the distribution of these cases by year, as well as the population of admitted individuals, the applications-to-place ratio, and the odds of admission, by field and by type of application.

**Table 1:** Applications and admissions, application/admissions ratio, and odds-ratio of admission—total population, by field study, and by type of application—2019–2023.

Year	2019	2020	2021	2022	2023	Total
Applications and admissions—total						
APP	106,545	107,531	103,699	85,613	89,407	492,795
ADM	8,352	8,301	8,113	7,776	8,055	40,597
APP/ADM	12.8	13.0	12.8	11.0	11.1	12.1
OR	0.078	0.077	0.078	0.091	0.090	0.082
By field of study						
Health and nature sciences (HNS)						
APP	47,223	47,852	48,886	40,198	39,945	224,104
ADM	1,946	1,914	1,881	1,839	1,879	9,459
APP/ADM	24.3	25.0	26.0	21.9	21.3	23.7
OR	0.041	0.040	0.038	0.046	0.047	0.042
STEM						
APP	22,314	22,431	20,022	16,854	18,431	100,052
ADM	3,012	2,971	2,857	2,753	2,792	14,385
APP/ADM	7.4	7.5	7.0	6.1	6.6	7.0
OR	0.135	0.132	0.143	0.163	0.151	0.144
Humanities						
APP	370,08	37,248	34,791	28,561	31,031	168,639
ADM	3,394	3,416	3,375	3,184	3,384	16,753
APP/ADM	10.9	10.9	10.3	9.0	9.2	10.1
OR	0.092	0.092	0.097	0.111	0.109	0.099
By type of application						
EP/PPI						
APP	27,387	29,570	33,418	23,447	26,547	140,369
ADM	2,235	2,886	3,189	3,021	3,513	14,844
APP/ADM	12.3	10.2	10.5	7.8	7.6	9.5
OR	0.082	0.098	0.095	0.129	0.132	0.106
AC						
APP	79,158	77,961	70,281	62,166	62,860	352,426
ADM	6,117	5,415	4,924	4,755	4,542	25,753
APP/ADM	12.9	14.4	14.3	13.1	13.8	13.7
OR	0.077	0.069	0.070	0.076	0.072	0.073

Source: FUVEST, 2019–2023.

The number of applications declined over the period, reaching its lowest level in 2022, while there was only modest variation in the number of admissions. The applications/admissions ratio ranged between 11 and 12 over the period, with an average probability of admission of 8.2% for the period as a whole, increasing over time. Careers in HNS account for the largest number of applications and the smallest number of places, resulting in an applications-to-place ratio that is significantly higher than in the exact sciences or the HUM. Careers in the humanities comprise the second-largest pool of applications, followed by STEM, a pattern observed in all years of the period analyzed. EP–PPI applications represented 28.4% of total applications over the period and 36.5% of admissions, whereas AC applications accounted for 71.5% of the total and 63.4% of admissions. Indicators of competitiveness suggest a higher applications-to-place ratio and lower probabilities of admission in Open Competition (*Ampla Concorrência*) relative to the EP–PPI quota track. Overall descriptive statistics therefore suggest significant differences in competitiveness and chances of entry across fields of study and across different types of application.

Table 2 presents descriptive statistics for the selected characteristics of interest used in the estimation of the models, also organized by field of study and by type of application:

**Table 2:** Selected socioeconomic characteristics of applicants and admitted students (mean values)—total population, by field of study and by type of application—2019–2023.

Variable	Total		By field of study						By type of application			
			HNS		STEM		HUM		EP/PPI		AC	
	APP	ADM	APP	ADM	APP	ADM	APP	ADM	APP	ADM	APP	ADM
Female	0.57	0.45	0.70	0.61	0.27	0.27	0.58	0.50	0.59	0.44	0.56	0.45
Race—black/pardo/native	0.21	0.22	0.21	0.21	0.20	0.23	0.22	0.21	0.36	0.41	0.15	0.10
Per capta income (LN)	7.64	7.82	7.60	7.73	7.69	7.79	7.67	7.88	7.11	7.36	7.86	8.08
Parents with HE	0.64	0.68	0.63	0.68	0.66	0.69	0.63	0.67	0.39	0.46	0.74	0.80
Labor force participation (40 h+)	0.10	0.14	0.07	0.08	0.09	0.10	0.14	0.22	0.16	0.18	0.07	0.12
Public basic education	0.33	0.39	0.32	0.38	0.32	0.43	0.34	0.36	1.00	1.00	0.06	0.04
Test score (0–90 scale)	46.46	59.28	47.08	59.30	48.64	61.33	44.36	57.54	41.15	52.92	48.58	62.96
*N*	492,795	40,597	224,104	9,459	100,052	14,385	168,639	16,753	140,369	14,844	352,426	25,753

Source: FUVEST, 2019–2023.

The description based on mean values of the selected variables reveals significant differences between applicants and admissions, as well as differences across fields of study and types of application. Women constitute the majority of applications (57%) but a minority of admissions (45%), a pattern observed across all fields except STEM, and across both types of application. They are particularly prevalent among applications in HNS (70%) and represent just over one quarter of applications in STEM. In terms of race, there is a balance in the proportion of black and pardo individuals between applications and admissions—suggesting no underrepresentation of black and pardo students among those admitted relative to applicants—although their share slightly exceeds 20% in all fields. Racial differences emerge more clearly across types of application, with higher proportions of black and pardo individuals among EP–PPI applicants, and a further increase in this proportion among those admitted. Per capita income is consistently higher among admissions, and admitted applicants in HUM display the highest average income levels. Income levels are lower among EP–PPI applications and admissions, well below the averages for the total population and for AC applications. Regarding family educational background, higher education attainment among parents or guardians is always more common among those admitted, across all fields (with little variation between them), although it is noteworthy that 64% of all applicants have at least one parent or guardian with higher education. Once again, more pronounced differences appear across types of application: only 39% of applications and 46% of admissions among EP–PPI applicants involve individuals with a parent or guardian holding higher education credentials, compared to proportions of 64 and 68%, respectively, for the total population. Labor market participation is higher among EP–PPI applications, although individuals engaged in regular work constitute a minority of the applicant population overall (10%), with a higher proportion among those admitted (14%), a pattern observed across all fields. In HUM, this proportion is higher, reaching 22% of all admissions, and lower in HNS and STEM, reaching at most 10% (among admitted individuals in STEM). The proportion of students coming from public basic education among applications (33%) is lower than among admissions (39%), suggesting an overrepresentation of students with this background among those admitted. This difference is largest in STEM (32% among applicants and 43% among admissions) and smallest in HUM (34 and 36%, respectively). Finally, proficiency as measured by the first-phase exam score is, as expected, always higher among admitted, with the largest difference observed in STEM. Overall, the descriptive results show pronounced differences in performance between EP–PPI and AC applications and admissions, and suggest that differentiation by type of application reduces the barrier posed by the standardized exam to chances of access.

The descriptive analyses illustrate the scope of the university’s admission process and allow it to be qualified in terms of its distribution across fields of study and careers, as well as in terms of differentiation by types of application. Over the period analyzed, more than 35% of places were allocated through quota policies, compared to a share of 28% of total applications, which signals advantages for EP–PPI students. The results also show relevant differences in the socioeconomic profile of those who apply and those admitted, systematically favoring admissions. Women constitute the majority of applications but a minority of admissions, a trend that is consistent across fields and types of application. The descriptive analysis further reveals significant differences in the socioeconomic profile of students competing through quota tracks, which indeed appear to be associated with applications from individuals with lower socioeconomic status, lower parental education, a higher proportion of black and pardo individuals, and greater participation in the labor market—indicating good policy targeting.

### 6.2 Multivariate analyses

In dialogue with the specialized literature, we delineated three working hypotheses that we seek to test with the proposed analytical design. We present the results below, organized around these hypotheses.

#### 6.2.1 Inclusion hypothesis

**Figure 1:**
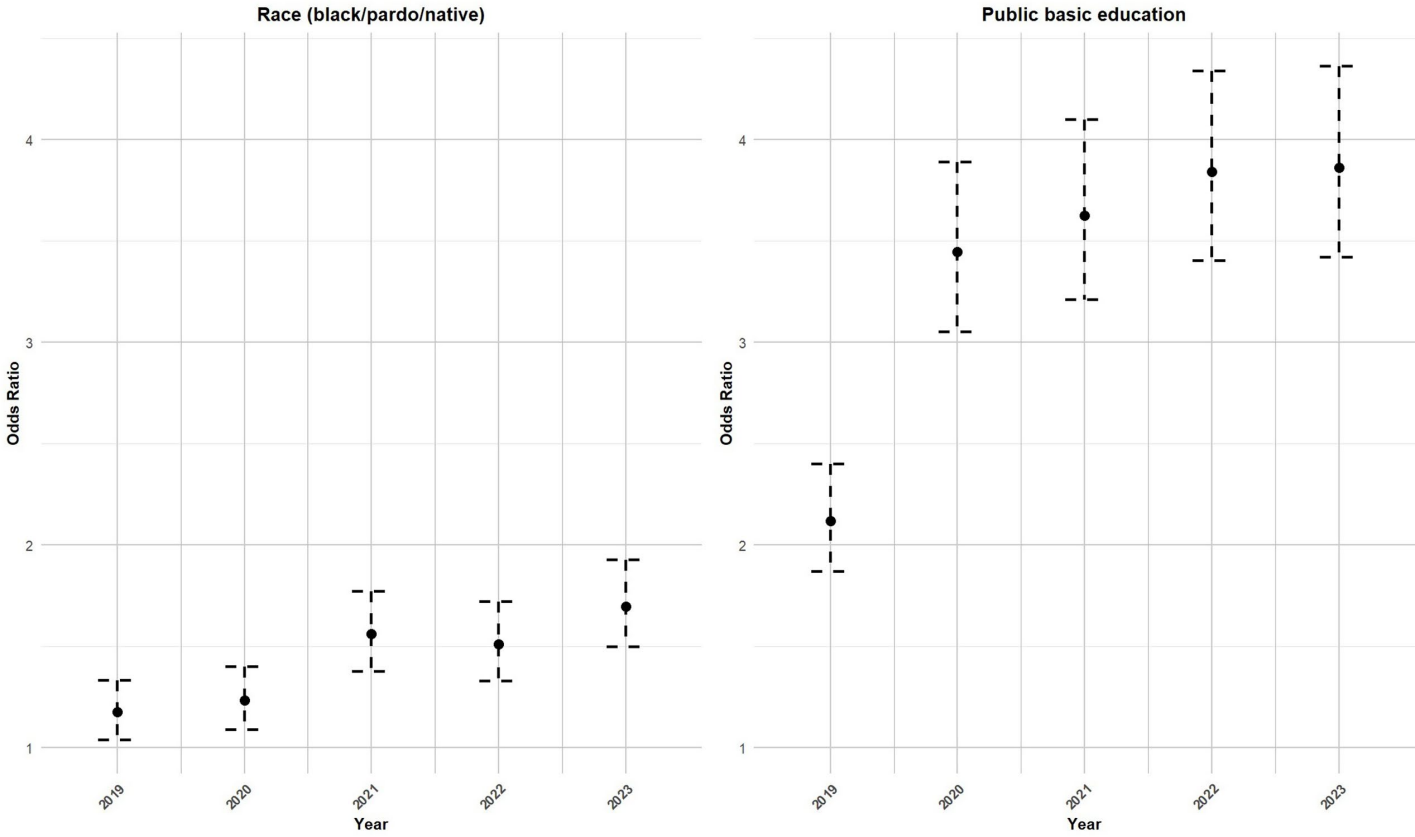
Estimated odds-ratios for admission—Race and public education—2019–2023.

In order to test the inclusion hypothesis, we estimate the specified model for the total population of applicants, focusing on the effects of the variables that identify the policy’s target populations—race and origin in public basic education. Our hypothesis posits that chances of admission for these groups are significantly higher and increase over the period analyzed. Figure 1 presents the results of the model estimates for these variables, expressed as odds ratios of admission:

Increasing coefficients in the panel graphs suggest characteristics that are increasingly associated with admission. The horizontal dashed line represents the significance test of the association between the variable under consideration and admission; the point indicates the observed effect estimate; and the dashed lines extending from the points represent the confidence intervals of the estimates. The coefficient estimates for the race variable show that, in all years, this variable is positively associated with admission, with a growing trend in the strength of this association over time. A similar pattern—though of greater magnitude—is observed for origin in public basic education. For this variable, the coefficient estimates are higher than those for race and display an even more pronounced upward trend in their association with admission. We can thus state that the chances of admission for graduates of the public school sector—which were already significantly higher than those of graduates from the private sector at the beginning of the period—became even more pronounced between 2019 and 2023. Overall, these results support confirmation of the inclusion hypothesis, by demonstrating that the characteristics defining the policy’s target population are significantly and positively associated with chances of admission during the period analyzed, particularly origin in public basic education.

#### 6.2.2 Segmented inclusion hypothesis

**Figure 2:**
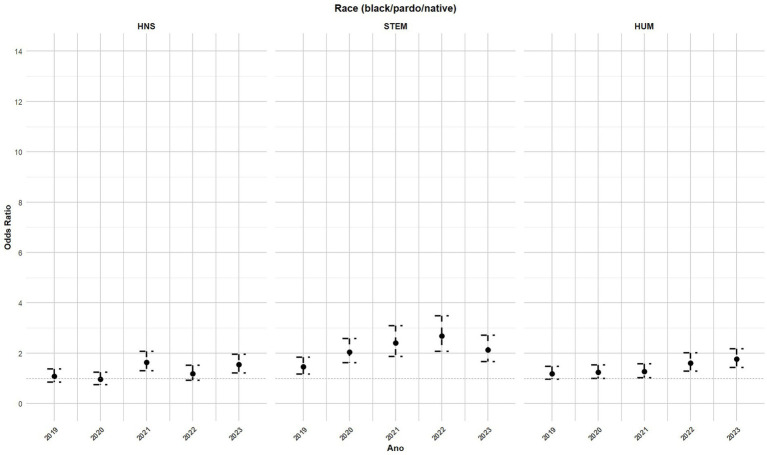
Estimated odds-ratios for admission—Race by field of study—2019–023.

The segmented inclusion hypothesis posits that the associations observed for the total population may differ depending on the field of study considered. In order to investigate this heterogeneity, we estimate the model for field-specific subgroups within each year and report, once again, the results observed for the variables defining the policy’s target population: race and origin in public basic education. Figure 2 below presents the estimated coefficients for the race variable, by year and by field of study (HUM, HNS, and STEM).

**Figure 3:**
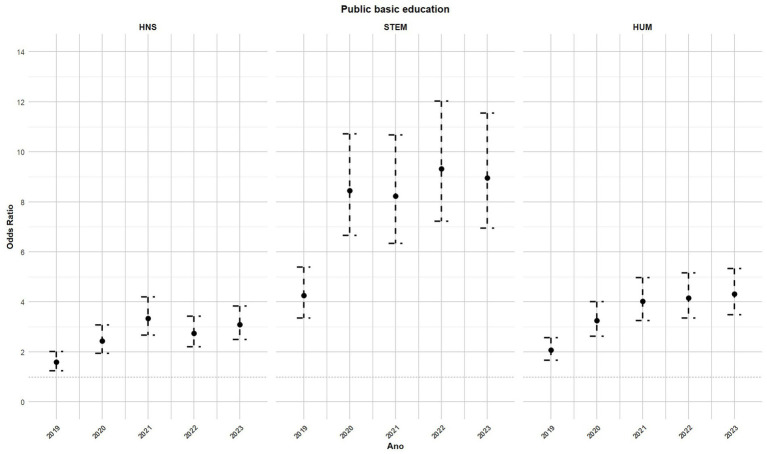
Estimated odds-ratios for admission—Public basic education by field of study—2019–2023.

The results show that the association between race and admission indeed varies depending on the field in which the applicant chooses to submit an application. In STEM, the association is unequivocal, with black and pardo individuals exhibiting significantly higher chances of admission in all years, with increasing estimates between 2019 and 2022. In HUM, the coefficients are significant from 2021 onward, but not in 2019 and 2020, when they are close to significance but cannot be considered statistically different from zero. For HNS, the evidence of association is less robust—significant in 2021 and 2023, but not in the other years. Figure 3 presents the results for the public basic education variable:

For the public basic education variable, the results are significant for all years and across all fields. The coefficient estimates for this variable are particularly high and display an upward trend, especially between 2019 and 2020. In STEM, the association is even stronger, reaching levels above those observed in the other fields. Overall, the results provide solid evidence that the 2019–2023 period is one in which students coming from public school have significantly higher chances of admission to USP through FUVEST entrance examination, controlling for the covariates included in the model estimation.

The results concerning the test of the segmented inclusion hypothesis suggest its partial confirmation. Indeed, the effects observed for the race and public basic education variables—generally positive, as shown by the results of the inclusion hypothesis test—indicate associations with admission that vary across fields of study. However, contrary to what the second part of the hypothesis anticipated, this association is more pronounced in STEM programs and careers, rather than in HNS and HUM. This finding points to counterintuitive processes of horizontal stratification relative to what has been documented in the specialized literature, favoring access by groups with lower average socioeconomic status to programs and careers associated with higher prestige and economic returns. In part, this may be explained by the fact that different fields began the period in 2019 with distinct levels of social selectivity in access—higher in STEM and HNS—and therefore offered greater scope for the expansion of access to the target groups of quota policies. In any case, the results provide evidence that the institutional design of reforms matters for shaping the structure of opportunities for access, and can even reverse long-standing historical trends related to stratification processes. This appears to be the case for the effects of the quota policy adopted by USP in the FUVEST selection process.

#### 6.2.3 Internal institutional differentiation hypothesis

**Figure 4:**
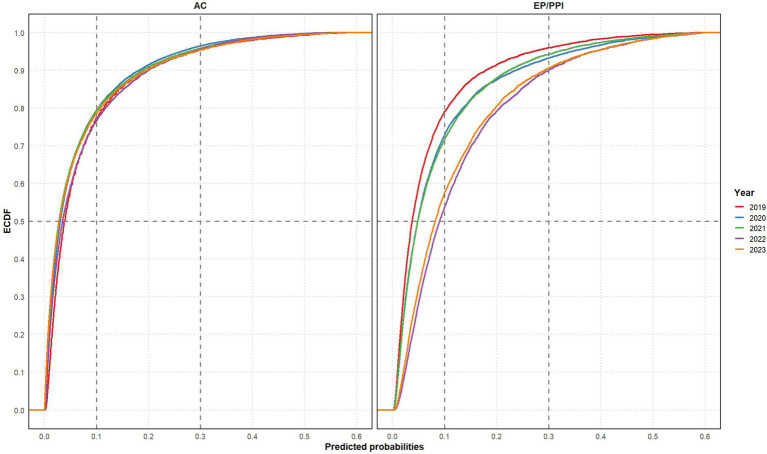
Empirical cumulative distribution function (ECDF) of admission probabilities—AC and EP-PPI applicants—2019–2023.

The internal institutional differentiation hypothesis posits that the differentiation of access mechanisms leads to the establishment of distinct opportunity structures. On this basis, we argue that processes of internal differentiation within institutions generate diverse patterns of stratification in access, and we consider the USP quota system to be well suited to illustrate and test this hypothesis. With our analytical design, we seek to provide comparative evidence on the distribution of chances of admission among groups of applicants who applied under different types of application (AC / EP–PPI). The internal institutional differentiation hypothesis proposes that the distribution of chances of admission will differ significantly across these groups. Figure 4 illustrates these distributions and their differences using empirical cumulative distribution curves.

The graphs in Figure 4 display the model-estimated predicted probability of admission on the *X*-axis and the cumulative proportion of cases on the *Y*-axis, with probabilities ordered from lowest to highest. The horizontal dashed line on the *Y*-axis indicates the expected probability for the 50% of cases with the lowest chances of admission. The two vertical dashed lines indicate the cumulative proportion of cases with up to 10% (0.1) and up to 30% (0.3) predicted chances of admission according to the model. Accordingly, the further to the left the curves are located, the more concentrated the probabilities are at lower levels, and the less accessible the opportunity structure is for that group in a given year.

The probabilities predicted by the models are generally quite low, given the rarity of the event of interest—admission. However, when comparing the distribution curves across types of application, significant differences emerge. First, the distribution of probabilities for AC applications is very similar across years. The evidence suggests that the distributions of chances of admission among AC applications do not change significantly over time, signaling a notable stability in the opportunity structure for this group over the period analyzed. By contrast, the distributions observed among EP–PPI applications differ in identifiable ways across years, suggesting an increasingly accessible opportunity structure for this group. In these cases, probabilities are more clearly concentrated at lower levels in 2019, 2020, and 2021, and at higher levels in 2022 and 2023—which display very similar distributions to one another. The results presented in the panel show, for example, that for AC applications, throughout the entire period, approximately 80% had 10% or lower chances of admission. Among EP–PPI applications, in 2019, 2020, and 2021, between 70 and 80% had 10% or lower chances, and this proportion declines to 50% in 2022 and 2023. This indicates an effective reduction of barriers to admission for these populations, whose probabilities become increasingly concentrated at higher levels. Taken together, these results support confirmation of the internal institutional differentiation hypothesis, showing that the differentiation of admission mechanisms through the implementation of quota policies has promoted the establishment of distinct opportunity structures across types of application, effectively favoring the chances of admission for the policy’s target populations.

The results of the tests of the three working hypotheses show that the reform implemented in access mechanisms through quota policies has primarily promoted increase in the chances of admission for students from public schools, but also for PPI students—even without an increase in the proportion of these groups among applications. This pattern characterizes an effective reduction in access barriers and, therefore, the promotion of inclusion. However, these transformations in chances of access are not distributed homogeneously across fields of study and have favored, in particular, EP–PPI students who apply to STEM, although consistent effects are observable across all fields over the period, especially among students from public basic education. The analysis thus points to counterintuitive patterns of horizontal stratification that favor students belonging to the target population of quota policies. Finally, the internal differentiation of admission mechanisms has effectively promoted the establishment of distinct opportunity structures across different types of application. For EP–PPI applications, it became increasingly accessible, in contrast to a stable structure for AC applications. Distinct parameters of stratification across types of application have been observed over the period, underscoring the importance of analyzing institutional reforms for understanding inequalities of opportunity and suggesting the capacity of this policy to reverse established patterns of inequality reproduction.

## 7 Discussion and conclusion

In this article, we seek to advance (1) the development of an interpretation grounded in documental analysis of the sequence of access-democratization policies implemented by USP since 2007, and of their positioning within a national context of higher education expansion and normative changes in the definition of access mechanisms to public higher education in Brazil; and (2) the systematization and analysis of data used to test hypotheses regarding trends in social selectivity in the FUVEST entrance examination, taking into account the set of reforms implemented in recent years.

The international discussion on higher education expansion and its relationship to opportunity structures provides highly relevant conceptual elements for elaborating the access problem as proposed here. It clearly highlights how expansion processes generate distinct stratification parameters depending on the institutional forms they assume. The dynamics of expansion exhibit specific characteristics according to the degree of system differentiation and diversification—generally favoring access as systems become more diversified, while simultaneously consolidating processes of horizontal stratification. By contrast, expansion tends to be more closely tied to the intentionality of reforms when it is less dependent on the private sector and more strongly state-centered, as shown by international comparative studies on the topic. Our analysis shows how heterogeneous logics of opportunity structuring coexist within the same system—particularly in diversified systems—and, taken together, shape the general conditions of access and the systemic selectivity barriers that characterize tendencies toward either the reproduction or the overcoming of inequalities.

From this comparative perspective, the case of higher education expansion in Brazil appears particularly interesting. This is due to the set of reforms and public policies aimed at increasing access levels that accompanied and fostered the expansion process in the country. These policies were largely characterized by a democratizing intent—especially with respect to access to public higher education—as documented by a substantial body of literature on the implementation of these initiatives. Another strand of scholarship on Brazil has recently documented the effects of these democratization initiatives, showing that, although differentiated regimes of social selectivity operate in the public and private sectors, policies directed at the federal public sector have achieved relative success in reducing socioeconomic barriers to access, through the centralization of vacancies allocation processes and the adoption of quota policies based on Law 12.711/2012. Beyond this, the literature has shown how the overall structure of access opportunities in expanding higher education systems is a function of heterogeneous social forces that compete for access within institutional frameworks that define the conditions of competition—the consolidation of distinct access regimes in the public and private sectors in Brazil being clear evidence of such a process. From this perspective follows the relevance of analyzing the case of USP, situating it within a diversified system—as is the Brazilian case—in a socio-historical context marked by intense reforms in public-sector access mechanisms, which in turn inspired the institution’s adoption of a quota policy aimed at transforming the opportunity structure of access. Did it succeed? This is the basic empirical translation of the question that motivated this study.

We translated our analytical intentions into three working hypotheses, and the overall results indicate that a process of access democratization is taking place at USP, albeit with important nuances. In general terms, we confirm the *inclusion hypothesis*, as the chances of admission for black, pardo, and native students, as well as from students coming from public basic education, are significantly higher than for other applicants, with a growing effect over the period analyzed. This occurs even without an increase in the proportion of PPI and public-school applications, signaling a genuine rise in the chances of access for these populations. The test of the *segmented inclusion hypothesis* revealed counterintuitive trends relative to the specialized literature, showing that in the case of USP, increases in chances of admission for the policy’s target populations occurred primarily in STEM—an area that concentrates programs with higher prestige and competitiveness—when compared to HUM and HNS. This effect is observed mainly among students coming from the public school sector rather than among black, pardo, and native students. This finding suggests that horizontal stratification does not necessarily reinforce inequality, but may under specific institutional designs operate as a channel of inclusion into high-prestige fields. Finally, the test of the *internal institutional differentiation hypothesis* allowed us to demonstrate that the differentiation of access mechanisms represented by the adoption of quota policies led to the establishment of distinct opportunity structures across types of application, progressively reducing access barriers for students belonging to the policy’s target population as the implementation of quotas advanced.

The overall configuration of opportunity structures in a higher education system undoubtedly results from the operation of heterogeneous access regimes across segments, fields, and institutions. Through the study of the USP case, we seek to illustrate the relevance of mobilizing concepts such as diversification and differentiation in the interpretation of institutional processes that are specific to higher education institutions themselves, and how this type of analysis can contribute to understanding the heterogeneity of trends that shape broader patterns. Moreover, analyzing the relationship between reforms and opportunity structures at the institutional level allows for the production of more detailed evidence regarding the mechanisms on which this relationship is grounded, as well as its nuances and the emergence of unintended effects of reforms. In doing so, it underscores the continued relevance of questions concerning the overcoming or reproduction of inequalities for understanding the distribution of educational opportunities in modern societies. The case of USP thus illustrates how institutional design matters not only for expanding access, but for reshaping the very structure through which inequality is reproduced or disrupted.

## Data Availability

The data analyzed in this study is subject to the following licenses/restrictions: the datasets are not open access. Requests to access these datasets should be directed to fuvest@fuvest.br.

## Footnotes

1 This is a field that has developed significantly in recent years with the incorporation of administrative records into the analyses, enabling important advances in understanding the heterogeneity of the opportunity structure in HE access, particularly between the public and private sectors.

2 *Fundo de Financiamento Estudantil* (Student Financing Fund) and *Programa Universidade para Todos* (University for All Program) respectively, from here on referred to as their acronyms in portuguese (FIES and ProUni).

3 Approximately 75% of students and 85% of institutions.

4 *Exame Nacional do Ensino Médio* (National Secondary Education Exam), from here on referred to as its acronym in Portuguese (ENEM).

5 A regime in which credentials would have a fundamentally social, rather than technical, content.

6 The *Programa de Apoio a Planos de Reestruturação e Expansão das Universidades Federais* that could be translated as Support Program for Restructuring and Expansion of Federal Universities. We’ll use the acronym from now on.

7 The *Sistema de Seleção Unificada* or Unified Selection System.

8 *Programa de Inclusão Social da USP* (USP Social Inclusion Program).

9 From here on those categories will be referred to by their acronyms in Portuguese (AC, EP, PPI).

10 Available in https://leginf.usp.br/resolucoes/resolucao-no-7373-de-10-de-julho-de-2017/.

11 At the time of application, the applicant makes two main choices: the career (a category that aggregates small, cohesive groups of undergraduate programs) and the type of application (AC / EP–PPI). An applicant who chooses the career of veterinary medicine under the AC category competes only with other applicants who submitted applications for the same career and the same type of application.

12 The databases provided by FUVEST comprises a total of 494,781 observations for the period 2019–2023. The proportion of cases with complete information amounts to 99.5% of the total.
